# Regulation of Key Genes for Milk Fat Synthesis in Ruminants

**DOI:** 10.3389/fnut.2021.765147

**Published:** 2021-11-25

**Authors:** Tong Mu, Honghong Hu, Yanfen Ma, Xiaofang Feng, Juan Zhang, Yaling Gu

**Affiliations:** School of Agriculture, Ningxia University, Yinchuan, China

**Keywords:** ruminants, milk fat, regulatory factors, genes, synthesis

## Abstract

Milk fat is the most important and energy-rich substance in milk and plays an important role in the metabolism of nutrients during human growth and development. It is mainly used in the production of butter and yogurt. Milk fat not only affects the flavor and nutritional value of milk, but also is the main target trait of ruminant breeding. There are many key genes involve in ruminant milk fat synthesis, including ACSS2, FASN, ACACA, CD36, ACSL, SLC27A, FABP3, SCD, GPAM, AGPAT, LPIN, DGAT1, PLIN2, XDH, and BTN1A1. Taking the *de novo* synthesis of fatty acids (FA) and intaking of long-chain fatty acids (LCFA) in blood to the end of lipid droplet secretion as the mainline, this manuscript elucidates the complex regulation model of key genes in mammary epithelial cells (MECs) in ruminant milk fat synthesis, and constructs the whole regulatory network of milk fat synthesis, to provide valuable theoretical basis and research ideas for the study of milk fat regulation mechanism of ruminants.

## Introduction

The demand for high-quality milk is increasing yearly worldwide, and the quality of milk has become the focus of consumers' common concern (1). Milk quality is mainly influenced by factors such as herd genetic structure, reproductive performance and feeding management level, and is also closely related to the main components in milk. Milk fat is rich in important energy substances required by humans and is a major component in the production of butter and yogurt. As a major target trait in dairy breeding, milk fat not only affects the flavor of milk and the nutritional value of milk, but is also one of the core competitiveness marks of the dairy industry (2). Studies have shown that milk fat plays a crucial role in human growth and development (3), the content and proportion of milk fat required by different consumer groups vary with children having a higher requirement for milk fat during their growth. Since 95% of milk fat is composed of saturated long-chain fatty acids (LCFA), which is associated with cardiovascular disease (4), older adults and people with diabetes, obesity and cardiovascular disease are more likely to choose low-fat or non-fat dairy products (5). Overall, the milk fat content, quality and fatty acid composition of milk are particularly important for human health. Therefore, improving milk quality and production efficiency is a top priority. Usually, the synthesis of milk fat can be achieved by combining genetic improvement with good management, however, following the discovery of DNA and advances in molecular biology techniques, it is clear that manipulation of DNA transcription and post-transcriptional regulation is a powerful means of influencing phenotypic outcomes. In summary, to provide a valuable theoretical basis and reference for the study of ruminant milk fat regulation mechanism, we were reviewed in this manuscript based on the mainline from fatty acid (FA) synthesis to the end of lipid droplet secretion, this manuscript expounds on the complex regulation mode of key genes in mammary epithelial cells (MECs) of ruminant milk fat synthesis.

## Synthesis of Milk Fat in Ruminants

**Figure 1:**
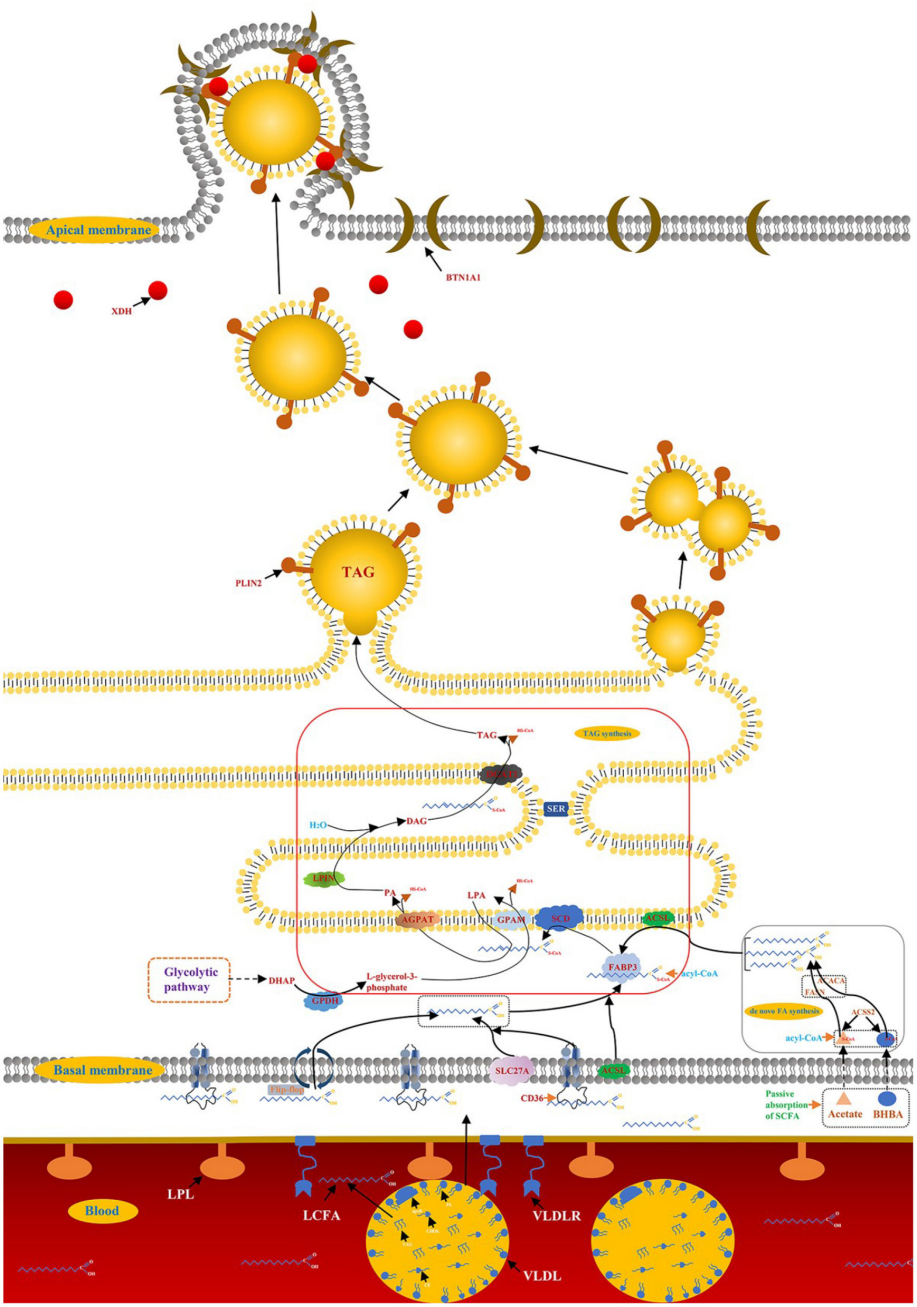
Synthesis of milk fat in ruminants. A detailed description of the model is reported in the manuscript.

Milk and dairy products are considered important the foods as early as 4,000 B.C., as evidenced by the petroglyphs of the Sahara Desert. Most scientific advances build the path of knowledge in very small increments, and the same is true of advances in lactation biology. The biology of bovine mammary lactation made great progress in the twentieth century, such as establishing the structure-function relationship between the MECs and the mammary gland, identifying the biochemical pathways of milk synthesis, and elucidating the role of hormones in the development and functional regulation of the mammary gland (6). Among these, milk fat synthesis, the formation and secretion of milk droplets are of particular interest, which affects the manufacturing characteristics and sensory quality of milk and dairy products (7). Early studies (1960–1980) in ruminants defined and quantified the main modes of mammary gland fat metabolism, including *de novo* synthesis of FA and LCFA uptake in the blood (6). The emergence of real-time fluorescence quantitative reverse transcriptase polymerase chain reaction (qRT-PCR) accelerated researchers' understanding of milk fat regulation, while high-throughput sequencing technology brought the research of milk fat synthesis into a new stage (8). Nearly 99% of the milk fat is the form of lipid droplets in the organism of ruminants, triglycerides (TAG) account for about 98% of the lipid droplets, and the remaining 2% are monoglycerides, diacylglycerol, cholesterol and free FA (9). Milk fat synthesis in ruminants is a complex molecular regulatory network, including *de novo* synthesis of FA, LCFA uptake in blood, LCFA transport and desaturation, TAG synthesis and lipid droplet secretion (10) (Figure 1).

## *De novo* Synthesis of FA

Acetic acid (acetate) and β-hydroxybutyric acid (BHBA) enter the cell membrane by passive diffusion in FA *de novo* synthesis, which is less than the flip-flop and protein-mediated uptake of LCFA (11). Acetyl-CoA carboxylase (ACACA), fatty acid synthase (FASN) and acyl-CoA synthetase short-chain family member 2 (ACSS2) were significantly up-regulated during lactation, and the *de novo* synthesis pathway of FA depended on these three key enzymes (12). Acetic acid and BHBA are activated in MECs by ACSS2 and converted to acetyl-CoA (acetyl-CoA) by binding to pyruvate. Acetyl-CoA is carboxylated by ACACA to form malonyl-CoA, medium-chain fatty acids (MCFA) or LCFA are synthesized under the action of FASN (carried out in cytosol) (13).

## Positive Regulation of *de novo* Synthesis of FA

### Acetate and BHBA Promote Milk Fat Synthesis

Acetate and BHBA are the main substrates for the *de novo* synthesis of FA in ruminants, which are essential for meeting energy requirements (70%) and milk fat synthesis of dairy cows (14). Research that nutritional strategies to increase rumen acetate uptake in dairy cows can increase milk fat through increase synthesis of FA (15). At the molecular level, the addition of different concentrations of acetate and BHBA increase the relative abundance of TAG content and lipogenic genes in bovine mammary epithelial cells (BMECs) *in vitro* (16), lipogenic genes include FASN, peroxisome proliferator-activated receptor α and γ (PPARA, PPARG) and phosphatidyl phosphatase 1 (LPIN1) (17). In addition, Acetate regulates milk lipid synthesis through the mTOR/eIF4E signaling pathway in BMECs (18). Thus, acetate and BHBA are not only the main substrates for *de novo* synthesis of FA in ruminants, but also have a significant positive regulatory role in milk fat synthesis.

### SREBP1

SREBP1 is synthesized as an inactive precursor in the endoplasmic reticulum and forms a complex with the SREBF partner (SCAP), which activates the adipogenic gene after being hydrolyzed in the Golgi apparatus and indirectly regulate milk fat synthesis (19). Silencing of SREBP1 reduces the mRNA abundance of ACACA and FASN in BMECs (40–65%) (20) and significantly down-regulates gene expression levels of ACSS2 (21), which is partly dependent on the enhancement of rapamycin target protein (mTOR) pathway. The regulatory functions of SREBP1 were also confirmed in goat mammary gland epithelial cells (GMECs) and found that AKT serine/threonine kinase (AKT1) phosphorylation has a key role in SREBP1 by activating the mTOR pathway (22, 23). To sum up, SREBP1 has a positive regulatory effect on the *de novo* synthesis of FA in ruminants and depends on the activation of mTOR pathway.

### RPL8, Ubiquitination and LXR Regulate FA *de novo* Synthesis Through SREBP1

Genome-wide association analysis (GWAS) reveals an important SNP (g.-931G>T) in the promoter region of ribosomal protein L8 (RPL8) in mammary tissue of lactating cows, which is strongly correlated with high expression of RPL8 (23). The expression levels of FASN, ACACA and SREBP1 in BMECs are significantly reduced when RPL8 is silenced (24). RPL8 may indirectly regulate FA *de novo* synthesis by down-regulating the expression of SREBP1 based on the positive regulatory effect of SREBP1 on FASN and ACACA (20), but the specific regulatory mechanism needs to be further verified. In addition, SREBP1 can be phosphorylated by recruiting glycogen synthase kinase GSK3 and then recruit ubiquitin ligase SCF-Fbw7 to be ubiquitinated and degraded by ubiquitin-proteasome system (UPS**)** when SREBP1 connects the target gene (25, 26). The expression of ACACA gene was significantly down-regulated after inhibiting the activity of UPS in BMECs, while the FASN gene was on the contrary (27). It is suggested that ubiquitin could regulate the expression of ACACA and FASN by regulating the transcription factor SREBP1 through the UPS pathway in the *de novo* synthesis of FA. Liver X receptor (LXR) is a member of the nuclear receptor superfamily, including LXR α and LXR β, which is widely expressed in lung, liver, brain and mammary gland (28). Gene expression of LXRα and SREBP1 was increased in a dose-dependent manner by different concentrations of T0901317 in GMECs, activation of LXRα also significantly increased the expression of FASN, ACACA and ACSS2, TAG content increased by more than 20% (29). In addition, SREBP1 activity and FASN gene expression were significantly upregulated in GMECs when the forkhead box protein O1 (Foxo1) gene was knocked down (30). Thus, LXR in the goat mammary gland positively regulates FA *de novo* synthesis in an SREBP1-dependent manner, which may be partially repressed by Foxo1.

### PPARs

PPARs, transcription factors, are activated by nuclear receptor super-family ligands, including PPARA, PPARG, and peroxidase proliferation-activated receptor δ (PPARD). PPARA and PPARG are expressed in liver and fat, respectively, while PPARD is widely expressed in fat, stomach, liver and lungs (31). PPARs have been reported to play important regulatory role in adipocyte differentiation, adipose metabolism and inflammation (32). PPARG positively regulates gene expression of FASN and ACSS2 in GMECs (33), which is consistent with the findings in buffalo and dairy cattle (34, 35). PPARD in rodents is mainly related to the catabolism of FA. The expression of PPARD at peak lactation is lower than that in dry period in the goat mammary gland. Overexpression PPARD does not affect the gene expression of ACACA and FASN, but inhibition PPARD significantly down-regulates the gene expression of FASN, which indicate that PPARD plays an important role in the dynamic balance of MECs in ruminants (36). In addition, PPARA activated by agonist WY-14643 in primary GMECs upregulated FASN expression by 1.1-fold (37). The above studies suggest a positive regulatory role of the PPARs family in FA *de novo* synthesis and find that the function of PPARD may be weaker than PPARG and PPARA. However, higher expression of transcription factors not play a significant role, vice versa. As mentioned above, the presence of WY-14643 agonist is more critical.

### THRSP

Thyroid hormone responsive spot (THRSP) is a key protein for new adipogenesis in non-ruminants. The expression of THRSP was the highest in subcutaneous fat of ruminants, the expression of THRSP during lactation was higher than that of in dry period in mammary gland (38). THRSP can positively regulates FASN in GMECs (39), however, ACACA activated by THRSP requires binding to spot-related protein (Spot14-R) (40). In addition, the positive regulatory effect of THRSP on the *de novo* synthesis of milk fat is inhibited by trans-10, cis-12 conjugated linoleic acid (t10c12-CLA) (41).

### CIDEA and CIDEC

The cell death inducing DFFA like effector A (CIDEA), a lipid droplet-related protein, is highly expressed in adipose tissue (42), which can promote liver lipid accumulation and the development of fatty liver (43). The expression level of CIDEA is high in mammary gland of lactating mice (44). Overexpression CIDEA and non-esterified fatty acid (NEFA) significantly up-regulated the expression gene of FASN and ACACA in BMECs and increased the content of TAG, the promoting effect of NEFA on milk fat synthesis disappeared after knockout of CIDEA, suggesting that high levels of NEFA promote FA *de novo* synthesis and milk fat secretion by stimulating CIDEA expression (45). Interestingly, interference RPL8 significantly upregulate the gene expression of CIDEA and decrease the mRNA abundance of FASN and ACACA, suggesting that the positive regulatory effect of RPL8 on FASN and ACACA may be stronger than the negative regulatory effect on CIDEA (24). Cell death-inducing DFF45-like effector c (CIDEC) is also a lipid droplet-associated protein located in lipid droplets and highly expressed in white and brown adipose tissue (46). CIDEC shares the same regulatory pattern as CIDEA and has been positively regulated ACSS2 (47). To sum up, both CIDEA and CIDEC play a positive role in the *de novo* synthesis of milk fat.

### Other Genes and Regulatory Factors

Glycosylphosphatidylinositol anchored high density lipoprotein binding protein 1 (GPIHBP1) is a member of the lymphocyte antigen 6 family and plays a key role in the transport and localization of lipoprotein lipase (LPL) (48). The expression level of GPIHBP1 is much higher in breast tissue than that in other tissues, there was a strong correlation between the SNP of the GPIHBP1 promoter region and milk fat rate (*P* = 5.0^E−18^) (23). After overexpression or interference GPIHBP1, the expressions of ACACA and FASN in BMECs are significantly up-regulated or down-regulated (49). Fatty acid elongase 6 (ELOVL6) plays an important role in transcriptional regulation of lipid metabolism and adipocyte proliferation in ruminants (50). Overexpression or interference ELOVL6 can promote the expression of FASN in BMECs and also has positive regulatory effects on insulin-induced genes (INSIG1 and INSIG2), SREBP1 and PPARG. So, we hypothesized that ELOVL6 could rely on transcription factors INSIG, SREBP1 and PPARG to regulate FASN (51). cAMP responsive element binding protein 1 (CREB1) is a member of the leucine zipper transcription factor family of DNA binding proteins, which is involved in the regulation of cellular lipid metabolism (52). The expression of CREB1 in rodents is closely related to the concentration of TAG (53). CREB1 overexpression significantly increases the mRNA abundance of SREBP1, ACACA and FASN in GMECs (54), which is similar to that in 3T3-L1 cells (55). Huang et al. (56) found that EGFR positively regulated FASN and ACACA in *de novo* synthesis of FA in GMECs through AKT and PLC-γ-1 pathways. LPL is the central factor in the hydrolysis of TAG and the uptake of free FA from plasma in very low density lipoproteins (VLDL) (57), the expression of LPL in early lactation is significantly higher than that in dry period, but the expression of LPL is relatively lower in peak and metaphase of lactation than that in early lactation. Knockout of LPL can reduce the expression of FASN, ACACA, PPARG and SREBF1 in GMECs (58).

## Negative Regulation of *de novo* Synthesis of FA

### t10c12-CLA

t10c12-CLA can reduce TAG storage and enhance FA oxidation (106), which is beneficial to human health (59). After 5 days of perfusion of t10c12-CLA (13.6 g/d) into the abomasum of dairy cows, the fat production capacity of dairy cows decreased by 82%. The mRNA abundance of FASN and ACACA also decreased by 40 and 39%, respectively (60), which was consistent with the results of adding t10c12-CLA to the diet of dairy goats (61). The decrease of milk fat caused by t10c12-CLA can be alleviated by adding palmitic acid (62). At the cellular level, 150 mol/L t10c12-CLA treatment of BMECs for 48 h significantly reduced MCFA and unsaturated fatty acid (USFA) content by 17.1 and 26.5%, respectively, and mRNA abundance of FASN and ACACA (63), this negative regulatory effect was accomplished in part by inhibiting SREBP1 activity (64) or activating the AMPK signaling pathway (65). This result was further confirmed by Zhang et al. (66). In summary, the inhibitory effect of t10c12-CLA on the *de novo* synthesis of milk fat in ruminants has been verified by a large amount of experimental data at both cellular and nutritional levels, and it is noteworthy that SREBP1 plays a key direct or indirect role in both milk fat inhibition and synthesis, further confirming the central position of SREBP1 in milk fat metabolism.

### LPS

Lipopolysaccharide (LPS), as the main component of the outer membrane of Gram-negative bacteria, is a key factor in inducing the release of pro-inflammatory cytokines (67). Previous studies have shown that LPS-mediated inflammation is associated with milk fat inhibition syndrome in lactating cows (68). Liu et al. (69) have shown that LPS can reduce the expression of FASN and ACACA in BMECs, which inhibit the *de novo* synthesis of FA. 14-3-3 γ is an important member of the 14-3-3 family and plays an important role in coordinating cell development (70). Overexpression 14-3-3 γ reduces LPS-induced cytotoxicity and improves cell survival. The expression levels of ACACA and FASN were significantly up-regulated in cells with high expression of 14-3-3 γ protein, the opposite results appeared after siRNA interference, suggesting that high levels of 14-3-3 γ protein reduce LPS-induced cell damage and promote milk fat synthesis by increasing cell viability and upregulating the expression of transcription factors related to milk fat synthesis (71). SREBP1 is not only the central regulator of milk fat synthesis (72), but also is involved in the adaptive response of breast infection and is important in the regulation of inflammation (73). Wang et al. (74) showed that the milk fat inhibition with time-dependent of BMECs induced by LPS was achieved by reducing the expression of SREBP1. The above results suggest that 14-3-3 γ can alleviate the milk fat inhibition of BMECs induced by LPS, and this inhibition depends on SREBP1.

### INSIG

Insulin inducible genes INSIG1 and INSIG2 are located in endoplasmic reticulum and play an important role in regulating lipid metabolism (75). The mRNA abundance of INSIG1 in breast tissue during peak lactation period was lower than that in dry period, which reveal the potential importance of INSIG in milk fat metabolism (76). After overexpression INSIG1 or INSIG2 in GMECs, the expression of ACACA and FASN was down-regulated, the content of TAG and total cholesterol (TC) were decreased. When INSIG1 and INSIG2 was interfered simultaneously, the contents of TAG and TC and lipid accumulation were significantly increased and ACACA expression was significantly up-regulated (77), It is shown that INSIG protein plays an important biological role in maintaining lipid homeostasis in goats, however, it is not known whether INSIG plays a similar function in milk lipid synthesis in cattle.

### AMPK and PTEN

AMP-activated protein kinase (AMPK) is a heterotrimeric energy-sensitive protein. Its activation reduces lipid synthesis in the liver tissues of many species (78). After BMECs was treated with AMPK activator, ACACA gene was inactivated, even though the expression of FASN increased, the *de novo* synthesis of FA decreased, suggesting that AMPK is more sensitive to ACACA (79). The latest research shows that activating AMPK can significantly increase phosphorylation of ACACA (144%), which further confirm that AMPK can inhibit the *de novo* synthesis of FA (80). In addition, sodium butyrate can relieve the inhibitory effect of AMPK on milk fat synthesis (81). Phosphatase and tensin homolog (PTEN) is a well-known tumor suppressor in non-ruminants, which regulates a variety of cellular processes through dephosphorylation of inositol phosphate substrates. Compared with the dry period, the mRNA abundance of PTEN decreased by 51.5% during the peak lactation period of goats, overexpression PTEN could significantly down-regulate the mRNA abundance of FASN and ACACA in GMECs (82), which suggest that there may be some mechanism in the mammary gland to reduce the expression of PTEN during lactation, thus weakening the inhibition on *de novo* synthesis of milk fat.

**Figure 2:**
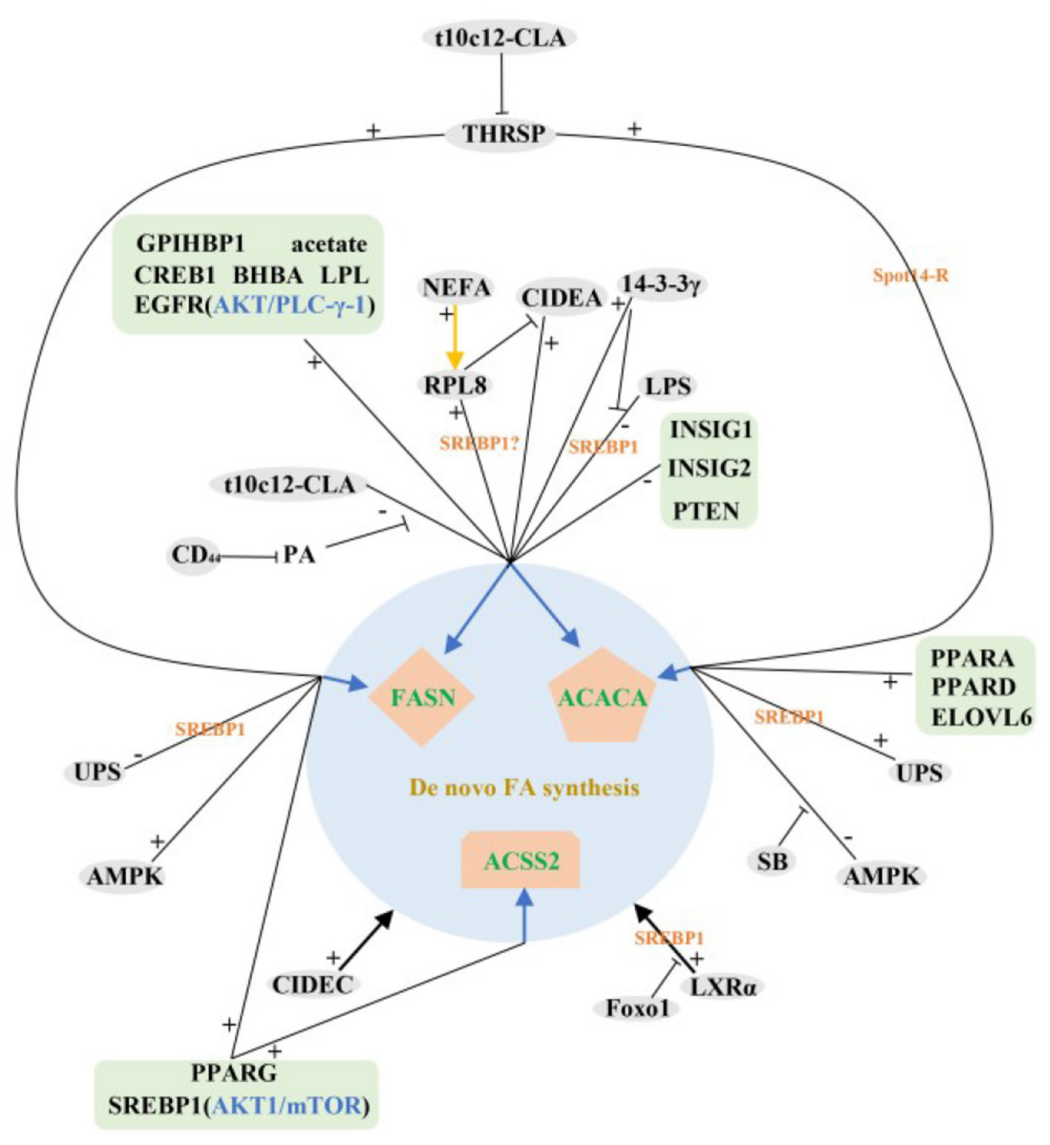
Regulatory network of *de novo* milk fat synthesis in ruminants. +: Positive regulation; –: Negative regulation; 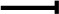 : Inhibition; 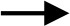: It can regulate FASN, ACACA and ACSS2 genes; 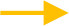: Regulate a gene.

In summary, most of the identified and validated regulatory factors so far have positive regulatory functions in the *de novo* synthesis of milk fat in ruminant livestock. Interestingly, the expression levels of these positive regulators were higher in lactation than that in dry period, while the negative regulators showed the opposite trend, suggesting that some mechanism may exist in ruminants to balance the expression abundance of positive and negative regulators during different lactation periods, the exact mechanism is still unclear. The above studies also fully confirm the overwhelming scientific evidence for the role of SREBP1 in the control of milk fat synthesis and repression, RPL8, ubiquitination, LXR, LPL, LPS and CREB1 play important roles in the control of SREBF1 expression. In addition, we find that mTOR/eIF4E, AKT/PLC-γ-1 and AMPK signaling pathways are pivotal in the *de novo* synthesis of milk lipids in ruminants (Figure 2).

## LCFA Uptake and Activation

*De novo* synthesis of FA and uptake LCFA from blood are the main ways of milk fat synthesis in mammals. For dairy cows in the first month of lactation, the intake of LCFA from the blood is dominant (6). Very low density lipoprotein receptor (VLDLR) cooperates with LPL to hydrolyze TAG in VLDL in blood and release LCFA (83). After entering the extracellular environment, LCFA is actively absorbed by MECs through fatty acid transferase (CD36) and fatty acid transporter (SLC27A), and another part of LCFA enters MECs through the mechanism of Flip-flop. The LCFA enters MECs through the above two ways will be activated by long chain acyl-CoA synthetase (ACSL) (84).

Previous studies have shown that THRSP has a positive regulatory role in the *de novo* synthesis of milk fat in goats, THRSP negatively regulates the expression of CD36 when LCFA is taken up from the blood, reflecting the dynamic balancing role of THRSP in milk fat synthesis (85). Adipose triglyceride lipase (ATGL) also negatively regulates the expression of CD36 (86), since ATGL is a key enzyme in the hydrolysis of TAG to produce diacylglycerol and free fatty acids and regulates lipid storage and release in adipocytes, ATGL may be more involved in the catabolism of milk lipids. The mRNA abundance of CD36 was down-regulated by 56% after interfering PPARA in GMECs, which was the same as the positive regulatory effect of PPARA in the *de novo* synthesis of milk fat, however, interfering PPARA up-regulated the mRNA abundance of ACSL1 and also significantly increased TAG content, suggesting a stronger negative regulation of ACSL1 by PPARA and further interpreting the dominance of LCFA uptake in the blood (37). Several studies have confirmed that t10c12-CLA, PPARG and PPARD exhibit positive regulation of both CD36 (35, 87, 106) and ACSL1 (34, 36, 63), however, Shi et al. (61) challenge the above view that t10c12-CLA A positively regulates CD36 and ACSL1, which may be related to factors such as the species, mode and amount of additives, the exact mechanism of action needs to be further verified by more in-depth experimental studies as well as large-scale populations. As previously described, 14-3-3γ, LXR, SREBP1 and CIDEC have positive regulatory roles in the *de novo* synthesis of milk fat, which perform similar functions during LCFA uptake (21, 29, 36, 69). The SLC27 family (SLC27A1 to SLC27A6) is considered to be bifunctional proteins with LCFA transport and acyl-CoA synthetase activity. SLC27A6 known as FATP6 and ACSVL2 is a transporter co-located with CD36 and mediates LCFA uptake (88). Knockdown of SREBP1 in GMECs significantly reduced SLC27A6 gene expression, and LXR agonists in turn upregulated SLC27A6 mRNA abundance, suggesting that LXR may be involved in regulating SLC27A6 gene expression in GMECs with an SREBP1-dependent manner (21, 29).

**Figure 3:**
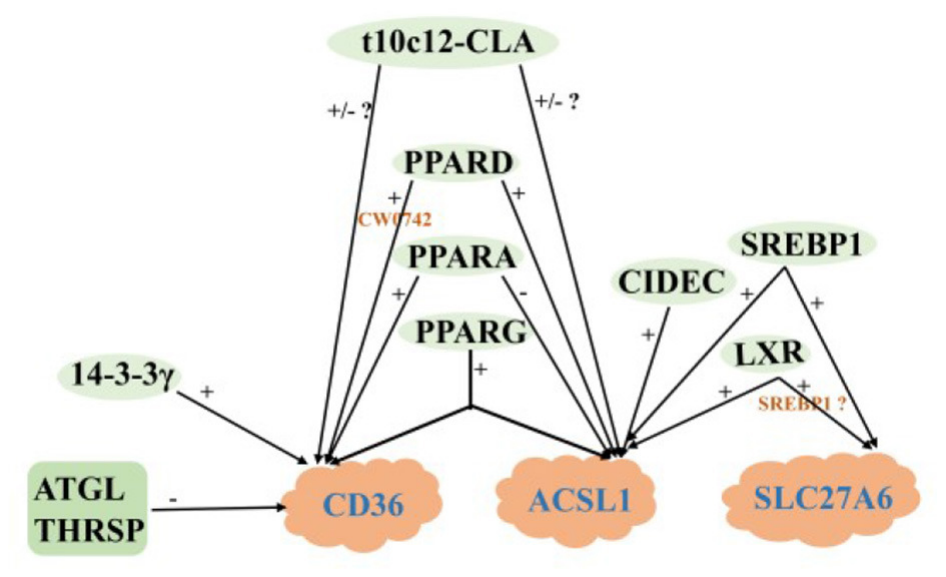
Regulatory network of LCFA uptake and activation in ruminants. +: Positive regulation; –: Negative regulation.

In a word, MECs involves many genes and key regulatory factors in the uptake of LCFA in blood, most of them have positive regulatory effects. At present, the mechanism of t10c12-CLA regulating CD36 and ACSL1 remain not clear, and the research on the regulation mechanism of ruminant SLC27A6 is still lacking (Figure 3).

## LCFA Transport and Desaturation

### FABP3 Is a Specific Transporter of LCFA

The activated LCFA requires specific transporters to diffuse quickly and freely in cells (89). Acyl coenzyme A binding protein (ACBP) is the main intracellular transporter of acyl-CoA in mammalian tissues (90), ACBP plays little role in milk fat synthesis in ruminants, which is supported by mouse data (91). Fatty acid binding protein (FABP) is a series of lipid chaperone proteins. The central cavity of the protein can bind LCFA and other hydrophobic ligands, which played an important role in LCFA uptake, transport, maintenance of cellular lipid homeostasis and regulation of fat storage (92). There are at least nine protein types of FABP in mammals. Except for FABP2, all FABP subtypes exist in the mammary gland of dairy cows. The mRNA abundance of FABP3 is the highest, and the expression of FABP3 is up-regulated during lactation (93). Therefore, the LCFA synthesized from *de novo* by FA and released from the blood mainly binds to FABP3 when activated in the cell (11).

AMPK is a heterotrimeric complex, which is composed of a catalytic subunit (α) and two regulatory subunits (β and γ). It regulates lipid metabolism mainly by modifying the transcription of adipogenic genes and participating in post-translational modification of key enzymes in lipid synthesis (78). The activation of AMPK can increase the expression of FABP3 and the oxidation of FA in muscle cells (94). After BMECs were treated with AMPK activator (600 μM AICAR), the expression of FABP3 and SREBP1 increased by 56 and 66%, respectively (79). However, reducing the proportion of mature SREBP1c and milk fat synthesis by 19% after AMPK activation, suggesting that AMPK may have a stronger effect on SREBP1c (80). Ubiquitin plays an important role in the *de novo* synthesis of FA in dairy cow mammary glands. When the activity of UPS was inhibited, the expression level of FABP3 and the content of LCFA were increased significantly (95). In addition, PPARD, PPARA and t10c12-CLA were also negative regulations of FABP3 (36, 37, 61), while LXR, SREBP1, RPL8, CREB1, ELOVL7, and PPARG were positively regulated of FABP3 (21, 24, 34, 54, 96, 97), and the regulation of LXR depended on SREBP1.

### Desaturation of SCD1

SCD1 is a rate-limiting enzyme in the biosynthesis of monounsaturated fatty acids (MUFA), which catalyzes the formation of Δ 9 unsaturated carbon double bonds (C16:1 or C18:1) (98). It is also a key enzyme in the endogenous synthesis of t10c12-CLA (99).

Identified Key transcription factors to regulate lipid synthesis including PPARs (100), SREBP (101), and LXR (102). PPARG was positively correlated with SCD1 expression in the mammary gland of goats during peak lactation (103), and overexpression PPARG1, PPARG2, and PPARA significantly upregulated SCD1 expression (37), which was also confirmed in cattle (34). Overexpression of SREBP1 enhanced the expression of SCD1 and its promoter activity in GMECs, while silenced SREBP1 reduced the expression of SCD1 and its promoter activity in GMECs (104), suggesting that SREBP1 regulates SCD1 expression at the transcriptional level. Meanwhile, LXRα can upregulate the mRNA abundance of SREBP1, and thus indirectly regulate the expression of SCD1 and promote the synthesis of MUFA and TAG in GMECs (29, 104). In addition, ELOVL6, EGFR and THRSP also have positively regulated SCD1. The regulation of SCD1 by EGFR depends on the AKT- and PLC-γ-1 signaling pathways (38, 51, 56).

After BMECs was treated with 150 mol/L t10c12-CLA for 48 h, the abundance of SCD1 decreased significantly (63). T10c12-CLA could also inhibit SCD1 expression by affecting the binding of SREBP1 protein to SRE and NF-Y sites (105), which was consistent with Zhang et al. (106) and Peterson et al. (64). Foxo1 can inhibit the participation of SREBP1 in lipid metabolism in the liver of mice. For dairy cows, Foxo1 also has a negative regulatory effect on the SCD1 (30). In addition, PTEN, INSIG1, and INSIG2 negatively regulate the expression of SCD1 in milk fat synthesis (77, 82).

**Figure 4:**
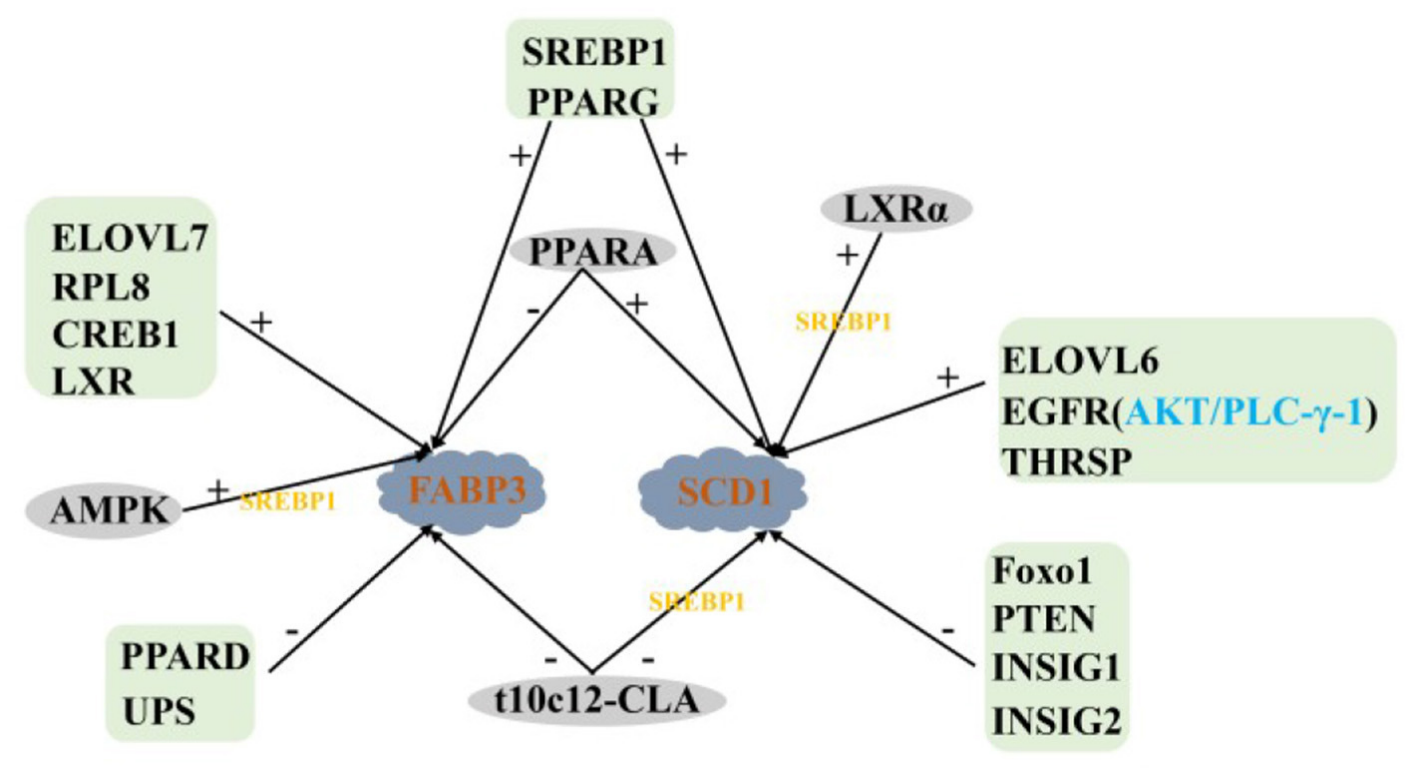
Regulatory network of LCFA transport and desaturation in milk fat synthesis of ruminants. +: Positive regulation; –: Negative regulation.

In summary, LXR, SREBP1, PPARG, PPARA and t10c12-CLA act together on FABP3 and SCD1. Among them, LXR, SREBP1 and PPARG positively regulate the expression of FABP3 and SCD1, while t10c12-CLA is the opposite. LXR participates in regulating the expression of FABP3 and SCD1 by SREBP1 (Figure 4).

## TAG Synthesis

Dihydroxyacetone phosphate (DHAP) produced by glycolysis produces glycerol-3-phosphate in the presence of glycerol-3-phosphate dehydrogenase (GPDH). A phosphate group was added to glycerol-3-phosphate under the action of glycerol-3-phosphate acyltransferase (GPAM), and then LCFA was activated to form lysophosphatidic acid (LPA). The LPA becomes the substrate of 1-acylglycerol-3-phosphate-O-acyltransferase (AGPAT) to form phosphatidic acid (PA). Phosphatidyl phosphatase (LPIN) catalyzes the formation of 1,2-diacylglycerol (DAG) from PA, and adds a third LCFA through DGAT1 to form a TAG molecule.

### Positive Regulation of TAG Synthesis

AGPAT6, DGAT1, LPIN, and GPAM are four key enzymes that regulate TAG synthesis in the endoplasmic reticulum of mammary glands. Their expression levels in the mammary glands of lactating cows are significantly higher than that in dry period dairy cows (12). LXR and CREB1 positively regulate the transcription of AGPAT6, DGAT1, LPIN, and GPAM genes in GMECs, and the regulatory role of LXR is dependent on SREBP1 (29, 54). PPARG positively regulates the expression of LPIN1, AGPAT6, and DGAT1 in buffalo and goat mammary glands (35, 107, 108). The expression of AGPAT6 and DGAT1 remain upregulated after overexpression of PPARG1, PPARG2 (33), and PPARA (37), respectively, in GMECs, PPARG1 is more abundant in mammary tissues, suggesting that PPARG1 may be a more important isoform in ruminant TAG synthesis. In contrast, PPARD knockdown increase the expression of AGPAT6 and DGAT1 and TAG content in GMECs, which is consistent with the negative regulatory effect of PPARD on the FABP3 gene (36). Specific protein 1 (SP1) is a zinc finger transcription factor, the expression abundance of AGPAT6, LPIN1, and DGAT1 were significantly decreased after silencing SP1 in GMECs. However, the expression of AGPAT6 and LPIN1 did not change significantly when SP1 was overexpressed by adenovirus. The expression of DGAT1 is still significantly down-regulated, which suggest that the decreased expression of DGAT1 may be the main reason for the inhibition of lipid accumulation in MECs (109). In addition, the DGAT1 gene is positively regulated by CIDEC and RPL8 in BMECs (24, 47).

AKT serine/threonine kinase is the intermediate of phosphatidylinositol 3-kinase/AKT (PI3K/AKT) signaling pathway, which regulates a series of biological processes including glucose transport, glycolysis, cell survival, protein, and fat synthesis (110). Activated AKT can regulate the expression of adipogenic genes in animal liver and fat (111). The content of TAG is increased by phosphorylation AKT1 and SREBP1 upregulated by mTOR in GMECs. Inhibition AKT/mTOR signal pathway can down-regulate the expression of AGPAT6, DGAT1 and GPAM (22). It is suggest that AKT1 plays an important role in regulating GMECs TAG synthesis, which may be through the mTOR/SREBP1 axis. Shi et al. (112) showed that inhibition ELOVL6 in GMECs significantly reduced GPAM expression and TAG concentration, ELOVL6 similarly positively regulated GPAM and AGPAT6 expression in buffalo MECs, suggesting that ELOVL6 not only regulates fatty acid chain extension and is involved in the *de novo* synthesis of FA, but also has a positive regulatory role in TAG synthesis (51). In addition, the GPAM is also positively regulated by THRSP in GMECs (38).

### Negative Regulation of TAG Synthesis

Baumgard et al. (60) found that the expression of the AGPAT6 decreased significantly after infusion t10c12-CLA into the abomasum of dairy cows in 5 days, which consistent with Zhu et al. (113) interfering with FASN in GMECs. Previous studies have shown that INSIG1 can effectively block the proteolysis activation of SREBP family members (114) and play an important role in *de novo* milk fat synthesis in ruminants (50). Fan et al. (76) measured the protein abundance of INSIG1 in the mammary gland of buffalo during peak lactation and dry period, and found that the expression of INSIG1 in the peak lactation period was lower than that in the dry period, and the overexpression INSIG1 significantly decreased the expression of GPAM and AGPAT6. The opposite result was obtained after INSIG1 was knockout, which was also confirmed by Li et al. (77). The above data provide strong support for the key role of INSIG1 in regulating milk fat synthesis in ruminants.

**Figure 5:**
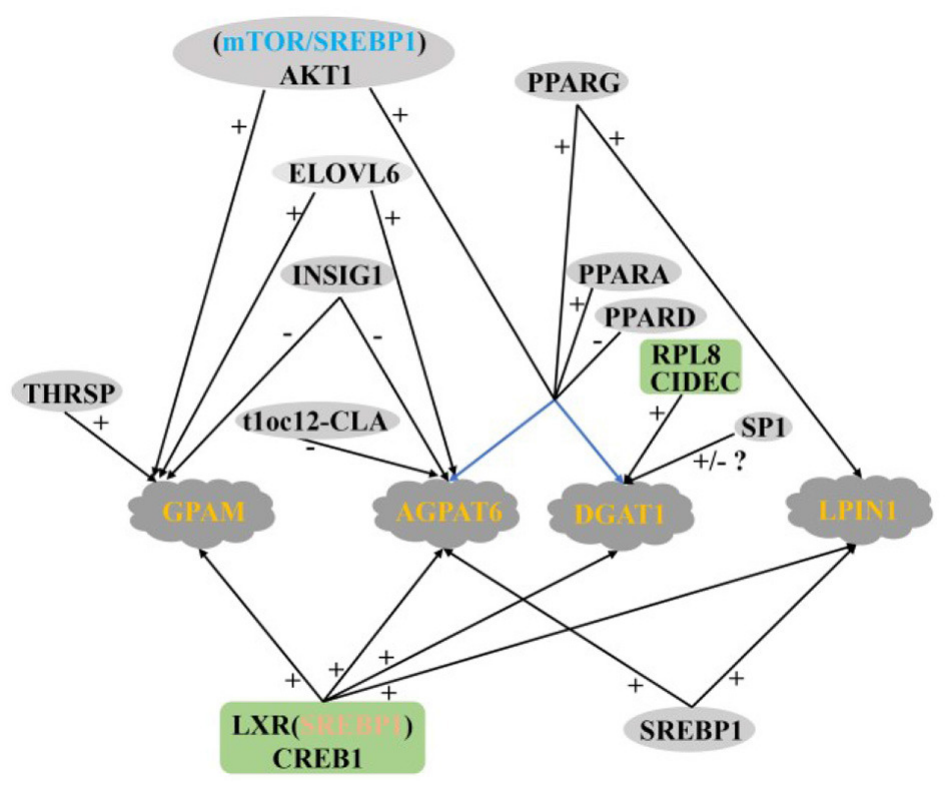
Regulatory network of key genes in ruminant MECs TAG synthesis. +: Positive regulation; –: Negative regulation.

In summary, the regulatory network of TAG synthesis in ruminant MECs is very complex, in which the PPARs family seems to play a very important role. Among the four key enzymes in TAG synthesis, there are still few reports on the regulation of LPIN and GPAM (Figure 5).

## Lipid Droplet Secretion

TAG forms between the two lobules of the endoplasmic reticulum and play an important role in the formation of lipid droplets. The extracellular secretion of lipid droplets is accomplished by the cooperation among adipose differentiation-associated protein (PLIN2/ADFP), xanthine dehydrogenase (XDH) and butyrophilin subfamily 1 member A1 (BTN1A1) (86).

### Regulation of PLIN2

Most of the milk fat produced during lactation is secreted into milk through the membrane wrapping process of cytoplasmic lipid droplets (115). PLIN2 is a cytoplasmic lipid droplet (CLD) binding protein (116). Shi et al. (117) found that overexpression PLIN2 can increase the lipid accumulation and TAG concentration of GMECs, and oleic acid (OA) supplementation can enhance its co-localization with CLD surface. In OA-free GMECs, PLIN2 knockout cannot eliminate lipid accumulation at the morphological level, suggesting that some other compensatory factors may assistant the formation of CLD. In summary, PLIN2 plays an important role in the formation of CLD in GMECs.

In non-ruminants, fatty acid elongase 5 (ELOVL5) is the key enzyme for the endogenous synthesis of unsaturated fatty acids (112). Compared with dry period and late lactation, the expression of ELOVL5 is the lowest in the goat mammary gland during the peak lactation, and the overexpression and interference of ELOVL5 positively regulate the expression of PLIN2. However, the content of TAG does not change, which may be due to an insufficient supply of substrates, finally affects the synthesis of milk fat *in vitro* (118). Previous studies have confirmed that SP1 is involved in the regulation of FA synthesis in humans (119), rats (120) and goats (121). Exogenous α-linolenic acid enhanced the binding of bovine SP1 to the proximal promoter of fatty acid elongase 7 (ELOVL7), resulting in the accumulation of lipid droplets in BMECs (122). After overexpression or interference SP1, the expression of PLIN2 decreased significantly (25%), and the content of lipid droplets secreted by GMECs decreased significantly by 9 and 8.5%, respectively (109). On the contrary, the expression of PLIN2 was significantly up-regulated after activation or knockout of PPARG in GMECs (107), indicating that other compensating factors are still a part of the regulation of PLIN2. In addition, PPARD, ATGL, and FASN positively regulate PLIN2 in milk fat synthesis of ruminants (36, 86, 113).

### Regulation of XDH and BTN1A1

Milk lipid droplets (LD) are the main components of membrane lipid secretion (123). When BTN1A1 was knockout, LD content decreased (124). In the process of secretion activation, XDH located in the apical membrane of MECs interacts with the cytoplasmic domain of BTN1A1 to regulate milk fat secretion (125). Compared with the dry period, the expression of XDH and BTN1A1 in the mammary gland of dairy cows was significantly up-regulated at the beginning of lactation (126). For different breeds, the expression level of XDH and BTN1A1 in mammary glands of lactating cows was significantly higher than those of yaks (12), which may also be one of the reasons for the high milk production of cows. Shi et al. (61) found that adding t10c12-CLA to the diet could significantly reduce the percentage of milk fat and the expression of BTN1A1 in dairy goats. Knockout/overexpression PPARG significantly down-regulated/upregulated the expression of BTN1A1 in buffalo (35). In addition, FASN, ATGL, CIDEA and LXR positively regulated the expression of XDH and Btn1a1 in ruminants (29, 45, 86, 113).

**Figure 6:**
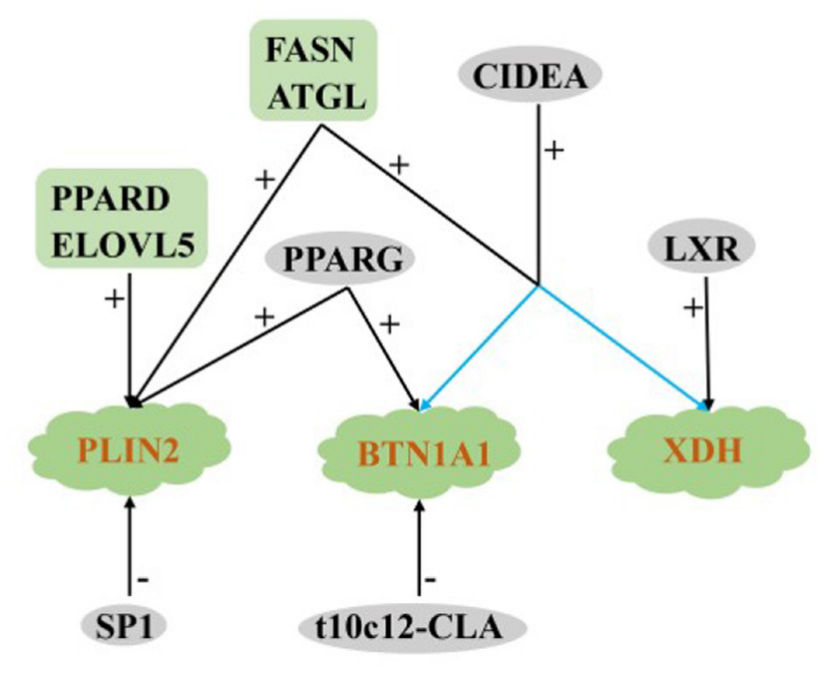
Regulatory network of key genes for MECs lipid droplet secretion in ruminants. +: Positive regulation; –: Negative regulation.

The above studies show that among the many regulatory factors, only ATGL and FASN have positive regulatory effects on the key genes PLIN2, XDH, and BTN1A1in lipid droplet secretion, reflecting their important role in fat droplet secretion in ruminants. Only SP1 and t10c12-CLA could significantly reduce lipid droplet secretion in ruminants, and other regulatory factors had positive regulatory effects (Figure 6).

## Summary

**Figure 7:**
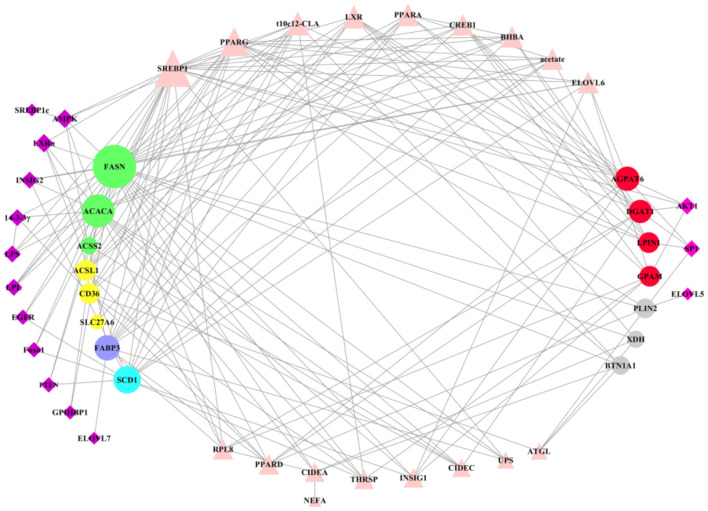
Overall regulatory network of key genes in milk fat synthesis in ruminants. The circle represents the key genes in milk fat synthesis in ruminants, of which the left is the key genes in *de novo* milk fat synthesis, LCFA uptake, FA transport and desaturation, and the right is the key genes in TAG synthesis and lipid droplet secretion. The quadrilateral represents the regulatory factors that regulate the key genes on the left and right sides of milk fat synthesis, respectively. Triangles represent the key factors that play a regulatory role in milk fat synthesis. The larger the figure, the more complex the regulatory relationship.

Most of many key factors have the positive regulation in milk fat synthesis, expression of these regulatory factors was higher in lactation than that in dry period, which may be a reflection of the balance of milk fat (Table 1). After clearly understanding the regulatory patterns of key genes in milk fat synthesis, we used the software Cytoscape3.7.2 to construct a network interaction map between all key genes and regulatory factors (Figure 7). Regulatory factors AMPK, LXRα, INSIG2, 14-3-3γ, LPS, LPL, EGFR, Foxo1, PTEN, CPIHBP1, ELOVL7, and SREBP1c have specific regulatory effects on key genes in the early stage of milk fat synthesis (*de novo* synthesis of FA, LCFA uptake in blood, LCFA transport and desaturation). Only AKT1, SP1, and ELOVL5 have specific regulatory effects in the later stage of milk fat synthesis (TAG synthesis and lipid droplet secretion). However, the regulatory effects of these specific regulators are not fixed, and, likely, some functions have not been explored yet, and more functions will be explored in the process of accumulating and updating the experimental data in the future to supplement or correct the existing data. In addition, SREBP1 plays a central role in maintaining essential transcription of genes related to milk fat synthesis, and PPARG, t10c12-CLA, and LXR also appear the critical importance. The results of t10c12-CLA regulation of CD36 and ACSL1 in LCFA uptake and activation remain controversial, which may be related to such as the species studied, the mode and the amount of additives. t10c12-CLA showed significant repressive effects in all other processes of milk fat synthesis. Both PPARG and LXR showed positive regulatory effects in milk fat synthesis, and LXR was involved in regulating milk fat synthesis in ruminant livestock with an SREBP1-dependent manner. It is noteworthy that there is a paucity of studies on the regulatory mechanisms of genes related to LCFA uptake and lipid droplet secretion in blood, and further in-depth investigation of them is necessary.

**Table 1:** Regulatory relationships of key genes for milk fat synthesis in ruminant livestock.

**Item**	**Key gene**	**Positive regulation factor**	**Negative regulation factor**
*De novo* synthesis of FA	ACSS2	SREBP1, LXRα, PPARG, CIDEC	
	FASN	SREBP1, RPL8, LXRα, PPARG, PPARD, PPARA, THRSP, CIDEA, GPIHBP1, ELOVL6, CREB1, EGFR, LPL	Foxo1, t10c12-CLA, LPS, INSIG1, INSIG2, AMPK, PTEN
	ACACA	SREBP1, UPS, LXRα, THRSP, CIDEA, GPIHBP1, CREB1, EGFR, ACACA	t10c12-CLA, LPS, INSIG1, INSIG2, AMPK, PTEN
LCFA uptake in blood	CD36	PPARA, PPARG, PPARD, 14-3-3γ	THRSP, ATGL
	SLC27A6	SREBP1, LXR	
	ACSL1	PPARG, PPARD, LXR, SREBP1, CIDEC	PPARA
LCFA transport and desaturation	FABP3	AMPK, LXR, SREBP1, RPL8, CREB1, ELOVL7, PPARG	UPS, PPARD, PPARA, t10c12-CLA
	SCD1	PPARG1, PPARG2, PPARA, SREBP1, LXRα, ELOVL6, EGFR, THRSP	t10c12-CLA, Foxo1, PTEN, INSIG1, INSIG2
TAG synthesis	AGPAT6	LXR, CREB1, PPARG, PPARA, SP1, AKT1, ELOVL6	PPARD, t10c12-CLA, INSIG1
	DGAT1	LXR, CREB1, PPARG, PPARA, SP1, AKT1	PPARD
	LPIN1	LXR, CREB1, PPARG, SP1	
	GPAM	LXR, CREB1, AKT1, ELOVL6, THRSP	INSIG1
Lipid droplet secretion	PLIN2	ELOVL5, PPARG, PPARD, ATGL, FASN	SP1
	XDH	FASN, ATGL, CIDEA, LXR	
	BTN1A1	PPARG, FASN, ATGL, CIDEA	t10c12-CLA

Overall, our survey of the literature shows that the regulation of milk fat synthesis in ruminants is controlled by a complex network of transcription factors and signaling proteins. With the factors involved in transcriptional and post-transcriptional regulatory networks are revealed, more precise interventions using transcription factor networks in combination with diet and feeding management may be more effective for milk quality improvement. It is also worthwhile to ponder that many important transcriptional regulators regulating milk fat synthesis in ruminants have been identified and confirmed in current studies, however, most of the findings seem to be isolated, and the order, strength and location of the regulators' roles in milk fat synthesis and the specific mechanisms by which MECs perceive an increase or decrease in milk fat remain unknown. Therefore, in future research, it is necessary for researchers to further explore the specific mechanism of regulatory factors and the interaction between them. Furthermore, milk fat synthesis in milk is a very complex regulatory mechanism, which not only including the simple expression of a relatively small number of genes encoding major proteins, but also is the product of complex interactions between multiple organs in the animal.

Therefore, the using of an integrated systems biology approach seems to be important for understanding milk fat synthesis.

## Author Contributions

TM: article writing. YM and HH: literature query and sentence modification. XF and JZ: article grammar modification. YG: conceptual analysis and writing-review and editing. All authors contributed to the article and approved the submitted version.

## Funding

This project was supported by the special breeding project of high-quality and high yield dairy cows in the Ningxia Autonomous region (Grant No: 2019NYYZ05).

## Conflict of Interest

The authors declare that the research was conducted in the absence of any commercial or financial relationships that could be construed as a potential conflict of interest.

## Publisher's Note

All claims expressed in this article are solely those of the authors and do not necessarily represent those of their affiliated organizations, or those of the publisher, the editors and the reviewers. Any product that may be evaluated in this article, or claim that may be made by its manufacturer, is not guaranteed or endorsed by the publisher.
